# Inhibition of Cyclin-dependent Kinase (CDK) Decreased Survival of NB4 Leukemic Cells: Proposing a p53-Independent Sensitivity of Leukemic Cells to Multi-CDKs Inhibitor AT7519

**DOI:** 10.22037/ijpr.2020.113170.14148

**Published:** 2020

**Authors:** Atieh Pourbagheri-Sigaroodi, Ava Safaroghli-Azar, Mehrnoosh Shanaki, Amir-Mohammad Yousefi, Ali Anjam Najmedini, Davood Bashash

**Affiliations:** a *Department of Hematology and Blood Banking, School of Allied Medical Sciences, Shahid Beheshti University of Medical Sciences, Tehran, Iran. *; b *Student Research Committee, Department of Hematology and Blood Banking, School of Allied Medical Sciences, Shahid Beheshti University of Medical Sciences, Tehran, Iran. *; c *Department of Medical Laboratory Sciences, School of Allied Medical Sciences, Shahid Beheshti University of Medical Sciences, Tehran, Iran.*

**Keywords:** Hematologic malignancy, Cyclin-dependent kinase (CDK), AT7519, Cell cycle, c-Myc; p53

## Abstract

An unbounded number of events exist beneath the intricacy of each particular hematologic malignancy, prompting the tumor cells into an unrestrained proliferation and invasion. Aberrant expression of cyclin-dependent kinases (CDKs) is one of these events which disrupts the regulation of cell cycle and subsequently, results in cancer progression. In this study, we surveyed the repressive impact of multi-CDK inhibitor AT7519 on a panel of leukemia-derived cell lines. Our data underlined that AT7519 abated the survival of all tested cells; however, in an overview, the response rate of leukemic cells to the inhibitor was varied irrespective of p53 status. Notably, the less sensitivity of leukemia cells to AT7519 was found to be mediated partly by the compensatory activation of c-Myc oncogene which was confirmed by the induction of a superior cytotoxicity upon its suppression in less sensitive cell. The blockage of cell cycle, as announced by induction of sub-G1 arrest as well as reduced S phase, resulted in a significant decrease in survival of acute promyelocytic leukemia (APL)-derived NB4 cells, as the most sensitive cell line, either as monotherapy or in combination with arsenic trioxide. Anti-leukemic effects of the inhibitor were further verified by apoptosis analysis, where we discovered that AT7519 induced apoptosis via alteration of pro- and anti-apoptotic genes in NB4. All in all, this study proposed that AT7519 is a rewarding agent opposed to APL; however, additional examinations should be performed to determine the advantages of this inhibitor in clinical setting.

## Introduction

Extensive genetic and experimental studies, which have converted into a better prospect for patients with hematologic malignancies, not only revolutionized our understanding of the molecules involved in the pathogenesis of the disease, but also changed the conventional chemotherapeutic approaches to the novel targeted therapies. A deep insight into history of cyclin-dependent kinases (CDKs), designated as ubiquitous cell cycle motors in charge of regulating cell growth and DNA replication, has unconcealed a road to the application of novel anti-cancer agents (1, 2). Great diversity among CDKs, their grave duty in the regulation of several intracellular functions, and frequent over-activation in a variety of human malignancies make them as broad dartboards being targeted by the arrows of cancer drugs in the field of targeted therapy (3, 4). By participating the panoply of novel CDK inhibitors in the army of promising therapeutic agents, the astonishing window of hope has been opened in front of cancer patients and their physicians. During past decades, highly selective- and pan-CDK inhibitors have allotted peculiar places to themselves in cancer treatment strategies and in this scenario, new nominees have joined in the parade of these inhibitors, which within them AT7519 is an appealing agent that has been entered into the top Phases of the clinical trials (5). 

Compared to the first generation selective CDK inhibitors , AT7519 is an amended inhibitor of CDK since it abrogates multiple CDKs and subsequently, halts cell cycle progression at several checkpoints (6, 7). Antineoplastic activity of AT7519 against various cancer types spanning from solid tumors to hematologic malignancies has been examined frequently and approved so far (8-11). Moreover, AT7519 co-treatment with different conventional chemotherapeutic drugs and inhibitors of oncogenic networks has also been surveyed by now, indicating an appealing capacity of AT7519 to synergize with different groups of anticancer agents (11, 12). Apart from the impressive effects in pre-clinical experiments, it has been reported that AT7519 exerted an extensive range of efficient cytotoxicity in xenograft mouse models (13, 14). A notable effect which has garnered more attention to the inhibitor is related to its tolerability and functionality in animal models, which makes this inhibitor a better choice among CDK inhibitors in clinical studies (15). The more the time passes, the more the doubts arise about the mechanism of action of AT7519. To the best of our knowledge, thus far, no study has explored the underlying mechanisms responsible for the less sensitivity of the hematologic malignant cells to AT7519 and our study proposed newly that the activation of c-Myc could outshine the efficacy of the inhibitor in leukemic cells. In addition, we found that AT7519 in combination with arsenic trioxide (ATO) provided an enhanced cytotoxicity against acute promyelocytic leukemia (APL)-derived NB4 leukemic cells; proposing this inhibitor as a promising agent in APL, either as a single agent or in a combined-modal strategy.

## Experimental

*Chemical agents and cell culture*

The main inhibitors AT7519 (multi-CDK inhibitor) and 10058-F4 (c-Myc inhibitor) were bought from Selleckchem (Munich, Germany). To make a stock solution, all ingredients were liquefied in dimethylsulfoxide (DMSO) and after that were separated equally, and kept at −20 ᵒC till utilization. Furthermore, arsenic trioxide (ATO) (SinaDaroo) as a conventional drug for APL treatment was used for expanded experiments. A panel of hematologic malignant cells including REH, Nalm-6, KMM-1, RPMI8226, U937, K562, and NB4 were chosen to survey the influence of cyclin-dependent kinase hindrance in leukemia. For drug treatment, aforementioned cells were cultured in suspension in RPMI 1640 medium supplemented with 2 mM l-glutamine, 10% heat-inactivated fetal bovine serum, and 1% penicillin and streptomycin in humidified incubator. The cells were exposed to the appropriate quantities of the inhibitors and equal amounts of solvents, as a control at the final concentration of 0.1%.

*Trypan blue exclusion assay*

The cells were seeded in 24-well plate in the culture medium and were treated with increasing concentrations of AT7519. After specified period of time, the pellets of the cells were re-suspended in serum-free complete medium and then, an identical amount of 0.4% trypan blue was added. The number of viable cells was enumerated manually and the viability percentage was calculated.

*Detection of apoptosis using flowcy tometry*

To explore the effects of AT7519 on the induction of programmed cell death, the cells were subjected to apoptosis analysis using annexin-V/PI staining. The experiment process has been described in our previous article (16).

*Analysis of cell distribution in the cell cycle*

The effect of AT7519 on the distribution of cells in different phases of cell cycle was examined by propidium iodide (PI) staining. After treatment of NB4 cells with the indicated concentrations of the inhibitor up to 48 h, cells were harvested, washed two times with 4 °C PBS in order to eliminate cellular waste, and later fixed in 70% ethanol overnight. Next, with the aim of DNA staining and RNA degradation, PI and RNase were added in their turn, respectively. Subsequently, the cells were incubated for further 30 min and the distribution of the cells was assessed by flow cytometry. The achieving data was interpreted using the Windows FlowJo V10 software.

*Metabolic activity analysis by using MTT test*

The cells were seeded in 96-well plates at a density of 5 × 10^3^ cells/well in 100 μL of culture medium with or without drug up to 48 h and kept in a humidified 5% CO_2_ incubator at 37 °C. At various time periods, the media was eliminated from each plate and MTT solution (5 mg/mL) was added to every single well in the plate which was incubated for 3 h at 37 °C. After we added 100 µL DMSO to the wells, the absorbance was measured at 570 nm. The percentage of the metabolic activity of the cells was determined by dividing the OD of a resulting formazan measured by an enzyme-linked immunosorbent assay (ELISA) reader in the drug-treated group by the control counterpart. 

*RNA extraction, cDNA synthesis and quantitative real-time PCR*

Total RNA from the cells was extracted using RNA Isolation Kit (Roche, Mannheim, Germany) and quantified by Nanodrop instrument. The reverse transcription reaction was performed using complementary DNA (cDNA) synthesis kit (Takara Bio, Shiga, Japan). cDNA was subjected to quantitative real-time PCR (qRT-PCR) and then, fold change values were calculated based on 2^−ΔΔCt^ relative expression formula. The detailed process has been described in our previous article (17).

*Determination of combination index and dose reduction index*

To investigate the efficacy of drug combinations, the reduction of cell survival was examined and the combination index (CI) and dose reduction index (DRI) were evaluated as described previously (18). The CI values of <1, =1, and >1 indicate synergism, additive effect, and antagonism of drugs, respectively.

*Acridine orange staining assay*

To investigate the contributory role of autophagy in AT7519 anti-leukemic effects on NB4, the cells were treated with different concentrations of autophagy inhibitor chloroquine (25, 40, 50 µM) and then washed three times with PBS. One μg/mL acridine orange (Merck, Darmstadt, Germany) was then added to each sample and after remaining for 15 min in the dark, the differences in acidity of autophagic lysosomes and cytoplasm/nucleolus were visualized under a fluorescence microscope (Labomed, Los Angeles).

*Statistical analysis*

The data were expressed as the mean ± standard deviation (SD) of three independent experiments. All presented data were analyzed using GraphPad Prism software using two-tailed student’s test and one-way variance analysis. In order to compare the control group and the drugs-treated ones, the Dennett’s multiple comparison test was used. A probability level of *P* < 0.05 was considered statistically significant.

## Results

*AT7519 exerted cytotoxic effect on a panel of hematologic cancer cell lines*

**Figure 1:**
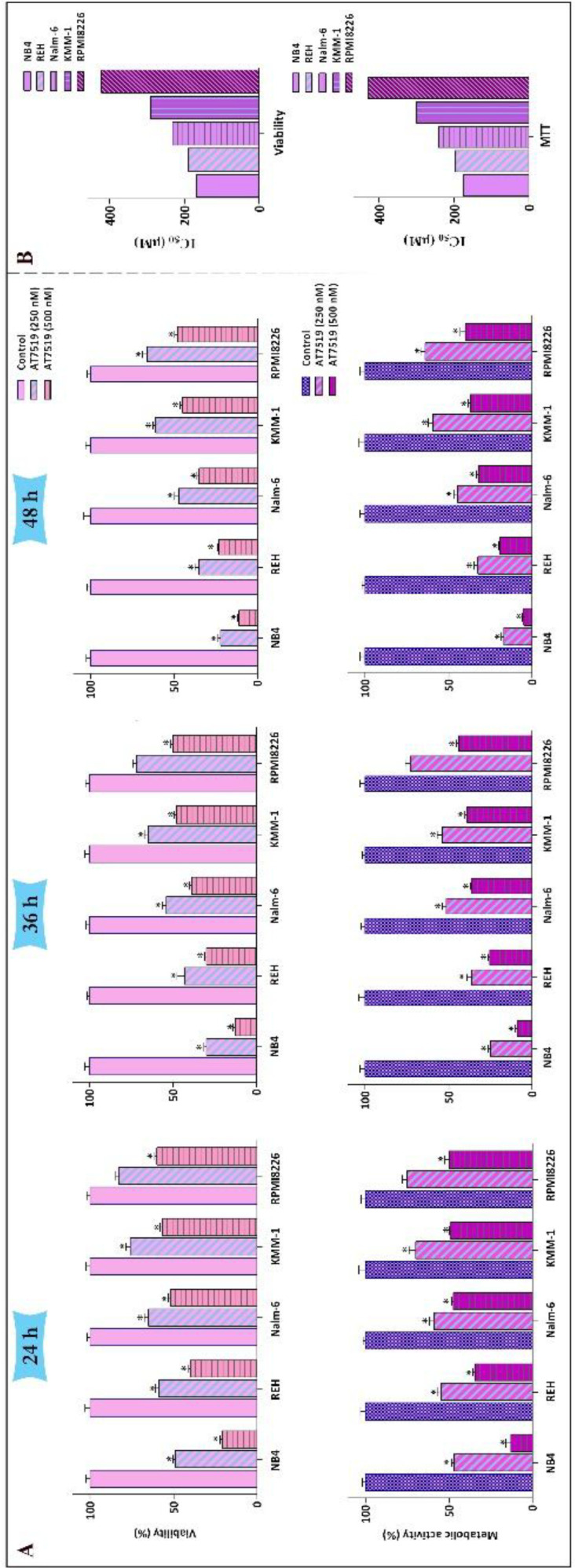
The anti-survival impact of AT7519 on a panel of leukemic cells. (A) Treatment of the cells with various concentrations of AT7519 abated both the viability and the metabolic activity in time- and concentration-dependent manners. (B) IC_50_ values were measured for two varied methods of survival assessment. Values are provided as mean ± SD of three separated tests. ^*^P ≤ 0.05 represents considerable alters from untreated control

Loads of studies have asserted that aberrant activation of cyclin-dependent kinases (CDKs) is correlated with tumor progression in various human cancers spanning from solid tumors to hematologic malignancies (19). To evaluate anti-cancer effects of a potent small molecule multi-CDK inhibitor AT7519 in hematologic malignancies, a group of leukemic cell lines including REH, Nalm-6, KMM-1, U937, RPMI8226, K562, and NB4 cells was exposed to the distinct concentrations of the inhibitor (250 nM and 500 nM) at several time intervals (24 h, 36 h, and 48 h). The resulting data from trypan blue and MTT assays demonstrated that not only AT7519 could reduce viability of all the aforementioned cells, also diminished the metabolic activity in time- and concentration-dependent manners (Figure 1A). Although the inhibitor exerted its anti-tumor effect on all tested cells, sensitivity of these cells was different to the inhibitor. As depicted in Figure 1B, following 24 h of treatment IC_50_ values for hematologic malignant cells were calculated for two different methods of survival assessment, and revealed that NB4 cell is the most sensitive cell line among all the tested cell lines.

*Leukemic cell sensitivity to the inhibitor is presumably correlated with c-Myc function*

**Figure 2:**
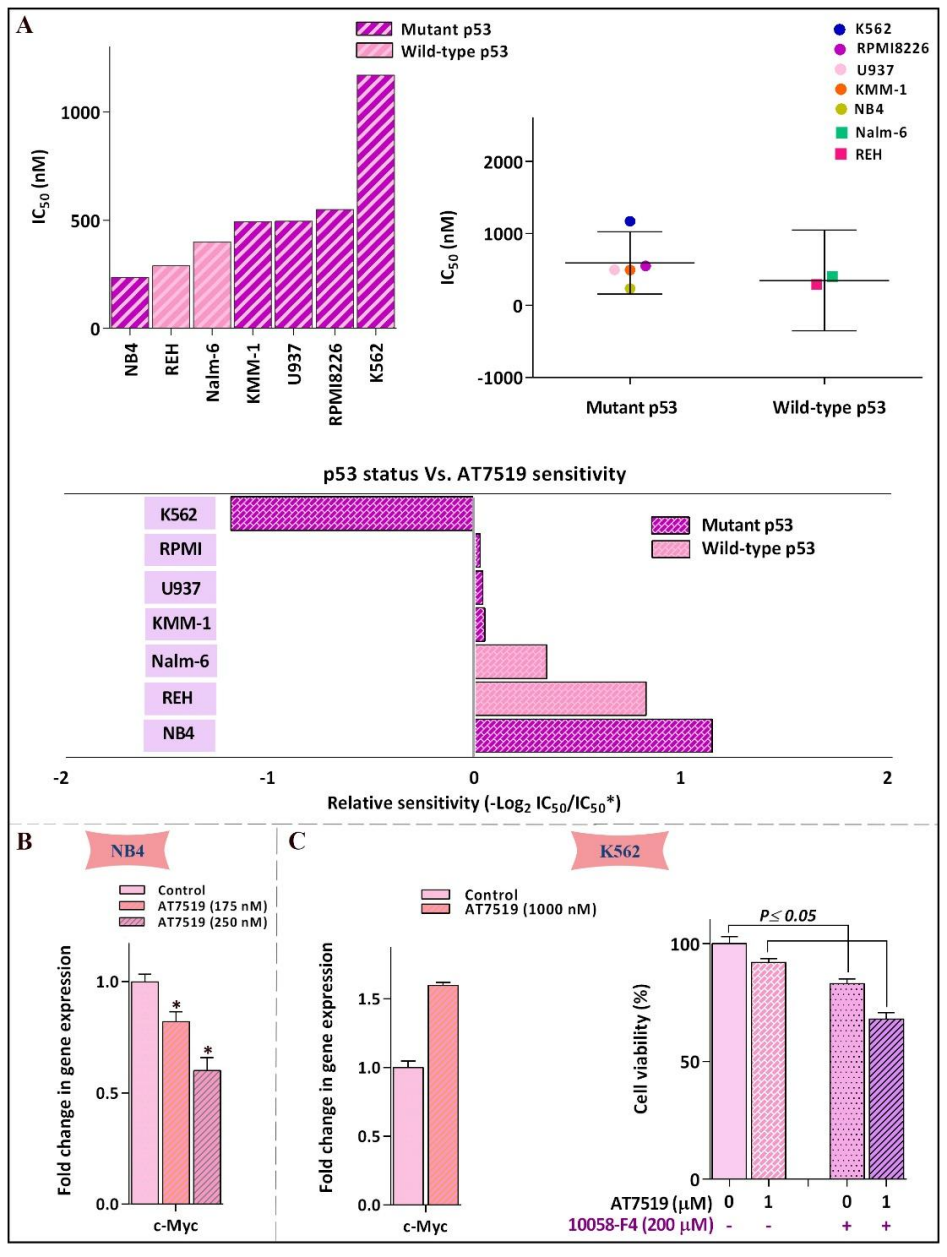
Leukemic cell response to AT7519 was irrespective of p53 status. (A) Relative sensitivity of all tested cell lines to AT7519. IC_50_ value of different leukemic cell lines to the inhibitor was determined using the formula: −log 2 (IC_50_ individual cell line/mean IC_50_ of all cells). There was no evidence of linkage between p53 status and cells sensitivity to AT7519. (B) AT7519 reduced the c-Myc expression in NB4 (C) but not c- in K562, as the less sensitive cell line. Values are provided as mean ± SD of three separated tests. ^*^P ≤ 0.05 represents considerable alters from untreated control

Multiple data sources reported that the deregulated c-Myc proto-oncogene, which serves as an important cell cycle regulator downstream of p53 tumor suppressor protein, could act as one of the most important regulator genes in charge of the genome instability, tumorigenesis, and evolution of therapeutic resistance (20). Hence, it made sense to hypothesize that the different leukemic cell sensitivity to the inhibitor could probably be mediated through either p53 or c-Myc expression. As announced in Figure 2A, correlation analysis of IC_50 _values among individual cell lines harboring either mutant or wild-type p53 demonstrated no significant link between molecular status of p53 and leukemic cell sensitivity to the inhibitor; proposing that AT7519 reduced the viability of the cells irrespective of p53 status and highlighted the potential application of this inhibitor in both wild-type and deﬁcient p53-expressing leukemic cells. On the other hand, the result of qRT-PCR showed that while cytotoxic effect of AT7519 led to a reduction in the transcription of c-Myc in NB4, an elevated expression level of the factor was found in less sensitive K562 cells (Figures 2B and 2C); proposing c-Myc as an important factor involved in leukemic cell response to AT7519. Accordingly, as presented in Figure 2C, although single agent of AT7519 induced minimal effect on the survival of K562 cells, co-treatment with AT7519 and small molecule inhibitor of c-Myc 10058-F4 resulted in a superior cytotoxicity as compared to each agent alone; highlighting the attenuating role of c-Myc in the anti-leukemic effect of the inhibitor. 

*AT7519 affected NB4 cell distribution in the different phases of the cell cycle*

**Figure 3:**
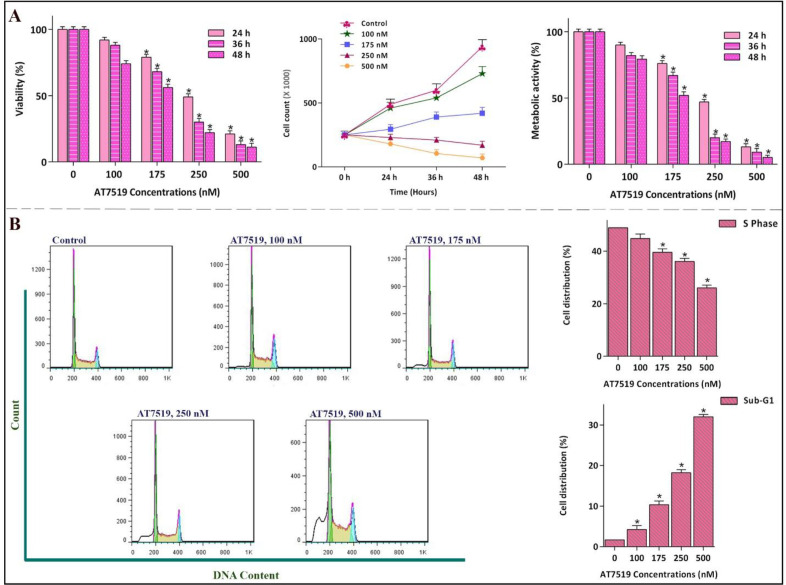
Anti-proliferative effect of AT7519 on NB4 and its impact on cell cycle progression. (A) the hindrance effect of AT7519 on CDKs was associated with a notable abatement in the viability and the number of viable cells in a concentration-dependent manner. (B) While the percentage of cells in S phase was lessened, cell population in Sub-G1 was escalated. Values are provided as mean ± SD of three separated tests. ^*^P ≤ 0.05 represents considerable alters from untreated control

To more precisely investigate the impact of AT7519 on the most sensitive cell line, NB4 cells were exposed to a broader range of AT7519 concentration and then, survival assays as well as cell cycle analysis were applied. Noteworthy, our results illustrated that the hindrance effect of AT7519 on cell cycle progression was associated with a notable abatement in the viability and the number of viable cells in a concentration-dependent manner (Figure 3A), which was in agreement with the decreased proportion of cell population in S phase of the cell cycle. As represented in Figure 3B, while the percentage of cells in S phase was decreased from 48.9% in the control group to 26% in the 500 nM-treated cells, cell population in sub-G1 was escalated approximately by 30-fold. 

*AT7519 induced apoptotic cell death in APL-derived NB4 cells*

**Figure 4:**
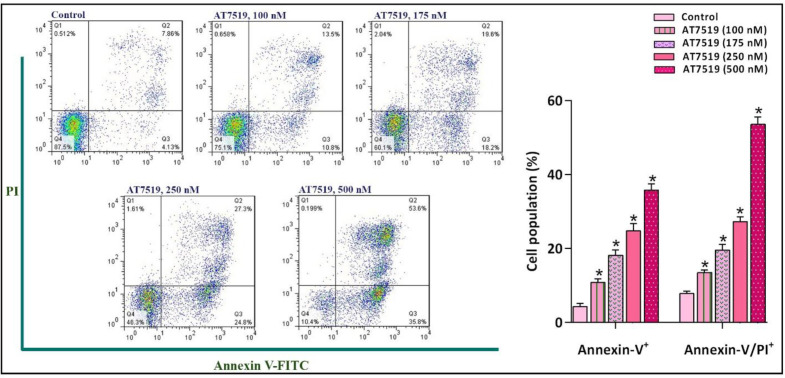
The suppression of CDK in NB4 cells was coupled with the induction of apoptotic cell death. FACS analysis of annexin-V/PI demonstrated that the inhibition of CDK using different concentrations of the inhibitor increased the proportion of both early and late apoptotic cells. Values are provided as mean ± SD of three separated tests. ^*^P ≤ 0.05 represents considerable alters from untreated control

An accumulating body of evidence has stated that the inhibition of CDKs gives rise to tumor regression via stimulating death-related signals leading to the apoptotic death in several sorts of cancer cells (21, 22). To investigate whether AT7519-induced cytotoxic effects were plausibly due to the apoptosis induction, the binding of annexin-V in combination with PI was analyzed by flow cytometry method. Intriguingly, FACS analysis of annexin-V/PI demonstrated that the inhibition of CDK increased the proportion of both early and late apoptotic cells, which was in agreement with the elevated sub-G1. As presented in Figure 4, AT7519 considerably increased annexin-V-positive NB4 cells from 10.8% in 100 nM to 35.8% in 500 nM of the inhibitor and also enhanced annexin-V/PI double-positive cell from 13.5% to 53.6% in 100 and 500 nM of the inhibitor, respectively. Taken together, our results showed that the inhibition of CDK using AT7519 halted NB4 cell progression, at least partly, through the induction of apoptotic pathway.

*Alteration of apoptosis-related genes upon treatment of NB4 cells with AT7519*

**Figure 5:**
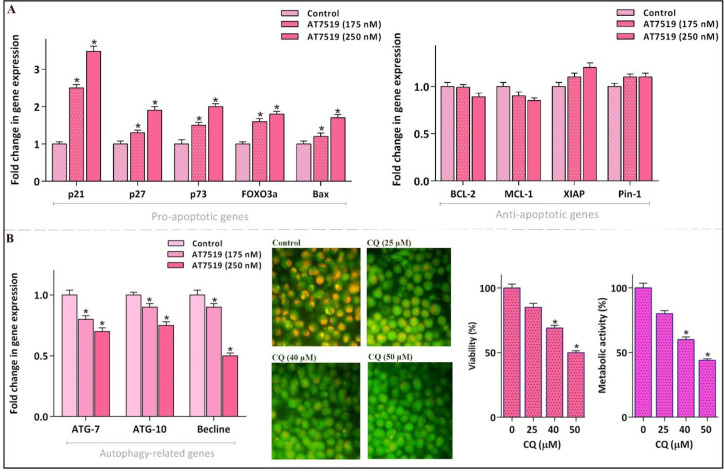
AT7519 could alter the apoptosis- and autophagy-related genes. (A) Although AT7519 up-regulated the mRNA expression levels of pro-apoptotic genes, it failed to significantly alter the transcription of the death repressor genes. (B) The inhibitor could remarkably reduce mRNA expressions of autophagy-related genes. The restriction of autophagy, as indicated by the decreased intensity of acridin orange, was coupled with the decreased survival of NB4 cell. Values are provided as mean ± SD of three separated tests. ^*^P ≤ 0.05 represents considerable alters from untreated control

To scrutinize the molecular mechanisms of action of AT7519 in NB4 cell line, inhibitor-treated cells were subjected to qRT-PCR analysis of a large cohort of genes involved both in pro- and anti-apoptotic pathways. The resulting data demonstrated that while AT7519 up-regulated the mRNA expression levels of death promoter genes, it could not significantly alter the transcription of death repressors (Figure 5A); hinting the point that AT7519-induced apoptosis in NB4 is probably mediated through the up-regulation of pro-apoptotic mediators. A great deal of data has shown that the most of CDKs have the ability to influence the cancer cells destiny under the correlation with a self-degradation pathway, autophagy (23, 24). In accordance, qRT-PCR analysis demonstrated that through treating NB4 cells with AT7519 the expression levels of autophagy-related genes were abated notably. To validate whether the suppression of autophagy is associated with an anti-survival effect in NB4, we investigated the effect of a well-known autophagy inhibitor chloroquine (CQ) in this cell line. Noteworthy, the inhibition of autophagy as revealed by the decreased intensity of acridine orange, was along with the lowering of NB4 cell survival which all in all suggested that presumably the cytotoxic effect of AT7519 is mitigated at least partly through activation of autophagy. 

*AT7519 potentiated cytotoxic effect of Arsenic trioxide (ATO)*

**Figure 6:**
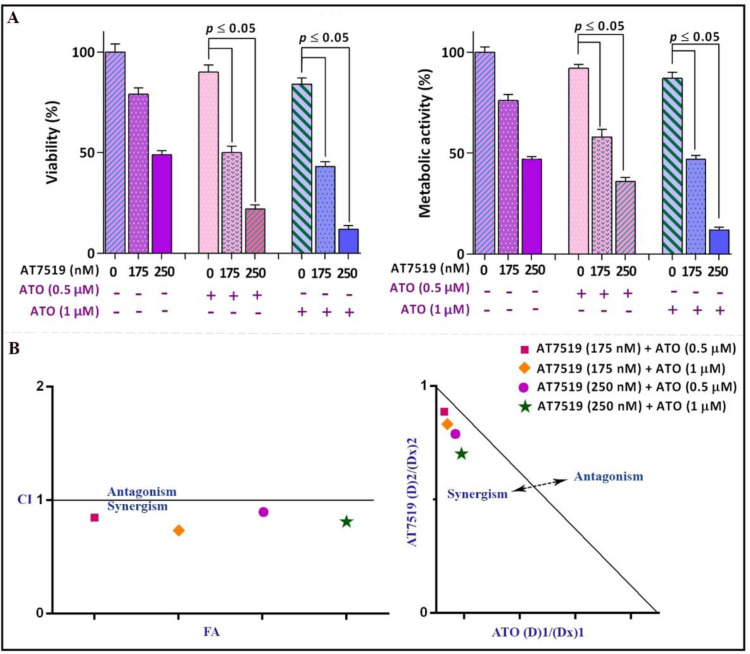
Surveying the synergistic impact of AT7519 and arsenic trioxide (ATO). (A) AT7519 could magnify the anti-leukemic influence of ATO in NB4 cells. (B) The results of both combination index (CI) and isobologram highlighted the synergistic effect between AT7519 and ATO. Values are provided as mean ± SD of three separated tests. *P* ≤ 0.05 represents considerable alters from untreated control

With reference to the prominent anti-cancer effects of the inhibitor as a single agent and its amplifying impacts on the chemotherapeutic drugs, it was enticing to assess whether AT7519 could boost the cytotoxic effects of arsenic trioxide (ATO), as a conventional medicine used in routine practice for APL treatment (12). Given this, NB4 cells were treated with the inhibitor either separately or in combination with the increasing concentrations of ATO. As shown in Figure 6A, the combination of AT7519 with ATO (0.5 µM and 1 µM) was more effective in inhibiting cell growth and survival as compared with either drug alone. To test whether the interactions between these drugs were synergistic or caused by an additive effect, the combination index (CI) was calculated. Determination of both CI and dose reduction index (DRI) values clarified that AT7519 has the ability to boost the cytotoxic effect of ATO in a synergistic manner (Figure 6B).

## Discussion

Great novel treatment strategies have been emerged in the arena of cancer therapy, which among them blocking the growth stimulatory signals proved to be a beneficial approach not only in tumor cells growth inhibition also in facing with drug-resistance. The crucial functions of cyclin-dependent kinases (CDKs) in the regulation of cell cycle together with the possibility to inhibit these kinases broadly escalates the universal passion for surveying the effectiveness of CDK inhibitors in human cancers (25). Multifunctional characteristic of these inhibitors coupled with their amazing ability to destroy proliferating cells have made them as propitious soldiers battling with cancer, which has long been a life-threatening disease sophisticated by endless mechanisms interfering with its treatment (26). In the present study, we found that the inhibition of CDK in a panel of hematologic cell lines using an ATP competitive multi-CDK inhibitor AT7519 decreased both the survival and the metabolic activity of all tested cells; however, in an overall comparison, the sensitivity of leukemic cells to the inhibitor found to be different among the cells. 

**Figure 7:**
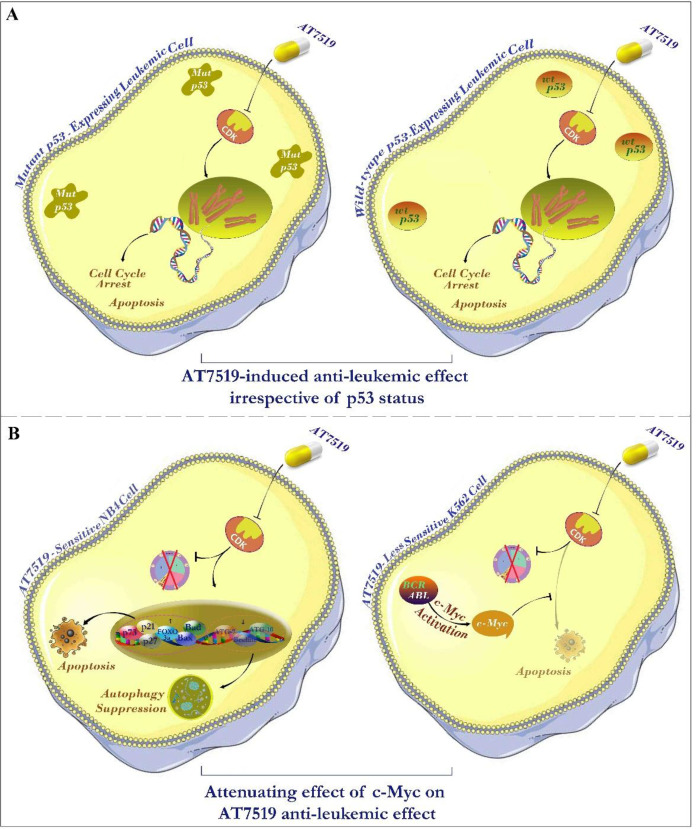
Schematic representation summarizes the resulting data of the present study. (A) Investigating the link between molecular status of p53 and leukemic cell sensitivity to the inhibitor hinted that AT7519 lessened the survival of leukemic cells independent of p53 status; suggesting the applicability of the inhibitor in both wild-type and deﬁcient p53-expressing leukemic cells. (B) The eminent anti-tumor impact of this inhibitor on leukemic cells was associated with the induction of apoptotic cell death through switching the balance between pro- and anti-apoptotic genes. The effectiveness of AT7519 may be probably eclipsed by the compensatory activation of c-Myc in AT7519 less sensitive K562 cells, proposing that co-targeting both c-Myc and CDK would be a better strategy in chronic myeloid leukemia (CML).

Mounting body of literature has described that the unrestrained activation of c-Myc oncogene, which is negatively regulated by p53, could overshadow the rate of cell response to the different drugs and be responsible for the acquisition of resistance to small molecule inhibitors of oncogenic signals (20). Accordingly, while the mRNA expression level of c-Myc was decreased in the most sensitive cell line NB4, we found that AT7519 treatment resulted in the induction of c-Myc expression in less sensitive K562 cells. In harmony, our results showed that the suppression of c-Myc in K562 increased the anti-leukemic impact of the inhibitor, indicating that the efficacy AT7519 could be eclipsed, to some extent, by c-Myc activation (Figure 7). Inconsistent, recent studies also highlighted the attenuating role of c-Myc on the cytotoxicity of small molecule inhibitors of PI3K in both acute myeloid leukemia and multiple myeloma cells (27, 28). Notably, investigating any plausible connection within molecular status of p53 and leukemic cell sensitivity to the inhibitor implied that AT7519 decreased the survival of leukemic cells independent of p53 status (Figure 7); shedding light on the applicability of the inhibitor in both wild-type and deﬁcient p53-expressing leukemic cells. P53-independent induction of cell death using small molecules targeting oncogenic molecules in leukemia cells have been also reported in previous studies (29, 30).

Our data also revealed that abrogation of CDKs, as announced by the induction of sub-G1 arrest coupled with a significant reduction in the S phase of the cell cycle, led to decreased survival in the most sensitive cell line (NB4) either as a single agent or in combination with arsenic trioxide (ATO). In a study conducted by Squires *et al.*, it has been illustrated that sub-G1 fraction of the cells in a panel of human tumor cell lines were escalated significantly upon treatment with a pan-CDK inhibitor (8). The interdependence between CDKs and apoptotic cell death has long been under extreme investigation and their tight relationship has been approved firmly (31). In this regard, a proportion of studies have stated that the inhibition of CDKs resulted in tumor relapse via affecting death-related signals leading to apoptosis in different types of human malignancies (32, 33). The results of our recent study also revealed that the inhibition of CDKs using AT7519, as revealed by the induction of G1 cell cycle arrest as well as the reduction of cyclins expression, resulted in decreased survival in acute myeloid leukemia (AML)-derived KG-1 cells (34). The beneficial anti-tumor effects of the inhibitor was verified by apoptosis analysis, where we discovered that suppression of CDK induced considerable apoptosis via switching the balance between pro- and anti-apoptotic target genes; insinuating the point that AT7519-induced cytotoxicity in NB4 is mediated, at least partly, through induction of apoptotic cell death.

The well-chronicled link between autophagy flux and cell cycle regulators has made this momentous stream as an outstanding target profitable for cancer therapy (35, 36). Aside from alterations in the expression of the genes involved in apoptosis, we found that hindrance of CDK was along with the impediment of autophagy-related genes. To investigate whether suppression of autophagy is associated with the anti-survival effects in NB4, we examined the effect of a well-known autophagy inhibitor chloroquine (CQ) in this cell line. Notably, we found that the inhibition of autophagy was coupled with decreased NB4 cell survival which altogether suggested that probably the cytotoxic influence of AT7519 is underrated partially through the activation of autophagy. In agreement, the results of recent studies revealed that the inhibition of autophagy either in acute myeloid or lymphoid leukemia cells was associated with a significant reduction in the survival of leukemic cells (27, 37). In conclusion, this study proposed that AT7519 is a rewarding agent opposed to acute promyelocytic leukemia, either alone or in a concomitant-therapy approach; though, additional examinations should be performed to determine the advantages of the inhibitor in the clinical setting.
